# Incidence, patterns, and factors associated with postoperative pulmonary complications in an ERAS cardiac surgery program: a 500-patient cohort study

**DOI:** 10.1186/s44158-026-00402-x

**Published:** 2026-05-08

**Authors:** Valentina Garelli, Christophe Abellan, Alexandra Othenin-Girard, Ziyad Gunga, Tamila Abdurashidova, Caroline Botteau, Estelle Brahier, Nathalie Parietti, Karima Alouazen, Valentina Rancati, Matthias Kirsch, Zied Ltaief

**Affiliations:** 1 The Department of Anesthesiology, Lausanne University Hospital and University of Lausanne, Lausanne, Switzerland; 2 The Department of Adult Intensive Care Medicine, Lausanne University Hospital and University of Lausanne, Lausanne, Switzerland; 3 The Department of Cardiac Surgery, Lausanne University Hospital and University of Lausanne, Lausanne, Switzerland; 4 The Department of Cardio-Respiratory Physiotherapy, Lausanne University Hospital and University of Lausanne, Lausanne, Switzerland

**Keywords:** Postoperative pulmonary complications, PPCs, Enhanced Recovery After Surgery, ERAS, Cardiac surgery, Cardiovascular surgery

## Abstract

**Background:**

Postoperative pulmonary complications (PPCs) remain the most frequent adverse events after cardiac surgery. Enhanced Recovery After Surgery (ERAS) programs aim to reduce postoperative morbidity, and data describing the incidence, patterns, and protective factors associated with PPCs within cardiac ERAS pathways remain limited.

**Methods:**

We analyzed 500 consecutive adult patients undergoing cardiac surgery within a standardized ERAS program at a tertiary center. PPCs included respiratory infection, respiratory failure, pleural effusion, atelectasis, pneumothorax, bronchospasm, or aspiration pneumonitis.

**Results:**

PPCs occurred in 130 of 500 patients (26%). The most frequent events were atelectasis (28.5%), respiratory failure (23.8%), and pneumonia (20%). Independent preoperative and intraoperative factors included age ≥ 70 years (OR 1.86, 95% CI 1.14–3.03), BMI ≥ 35 kg/m^2^ (OR 2.37, 95% CI 1.18–4.76), active smoking (OR 1.95, 95% CI 1.13–3.37), frailty (OR 2.21, 95% CI 1.12–4.33), and redo surgery (OR 3.09, 95% CI 1.52–6.26). Patients with PPCs had longer ICU and hospital length of stay, higher ICU readmission rates, and increased postoperative complications, without a significant difference in 30-day mortality. Exploratory analyses identified early extubation and shorter chest drain duration as variables associated with a lower incidence of PPCs, whereas postoperative delirium and transfusion were associated with an increased risk of PPCs.

**Conclusion:**

PPCs affected one in four patients in this ERAS cardiac surgery cohort and were associated with significant morbidity. Preoperative factors including age, obesity, frailty, and redo surgery were the main associated factors. Among these, frailty and active smoking emerged as key modifiable associated factors that remain incompletely addressed and represent important targets for optimization. Several postoperative factors, including early extubation, delirium, transfusion, and chest drain management, were potential targets for optimization. Further studies are needed to better clarify their causal role and to guide targeted interventions.

**Supplementary Information:**

The online version contains supplementary material available at 10.1186/s44158-026-00402-x.

## Background

Postoperative pulmonary complications (PPCs) are the most common adverse events after cardiac surgery, affecting up to 50% of patients [1, 2]. PPCs are associated with increased length of stay, morbidity, and mortality [3, 4]. Reported incidence and range vary widely across studies, reflecting heterogeneity in patient profiles, surgical procedures, perioperative management, prevention bundles, and mainly diagnostic definitions [2, 5]. Beyond baseline patient-related risk factors, perioperative management and prevention, including prehabilitation, anesthesia and ventilation strategies, postoperative analgesia, transfusion practices, and postoperative mobilization, play a key role in the development of PPCs [2, 6–8]. Enhanced Recovery After Surgery (ERAS) programs provide a structured, multidisciplinary approach to optimize perioperative care and reduce complications with multiple guidelines and expert consensus recommendations specifically targeting the prevention of PPCs in cardiac surgery [9]. Our cardiac surgery ERAS program was implemented in 2023 and combines standardized perioperative care, protective ventilation, early extubation and mobilization and is expected to impact the incidence and clinical course of PPCs [10]. The present study aims to describe and analyze the incidence, distribution, predictive factors and clinical impact of PPCs in the first 500 patients managed within an ERAS pathway for cardiac surgery at our institution.

## Material and methods

Our single-center study, carried out in compliance with the Declaration of Helsinki and following the STROBE reporting guidelines, received approval from the Ethics Committee of the “Commission cantonale d’éthique de la recherche sur l’être humain du Canton de Vaud” (CER-VD #2024-00632).

### Population

The Enhanced Recovery After Surgery (ERAS) program for cardiac patients was launched at Lausanne University Hospital in May 2023, with patient inclusion ongoing through December 2025. Adults aged 18 and older who underwent elective cardiac surgery via median sternotomy with cardiopulmonary bypass (CPB) were eligible, regardless of the procedure’s complexity or any prior history of cardiac surgery. Patients were excluded if they underwent urgent procedures, received a ventricular assist device, underwent heart transplantation, or declined consent for data use.

### ERAS protocols

Our ERAS organization, implementation and protocols were detailed previously [8, 10–12]. Briefly the cardiac surgery ERAS pathway is structured into sequential steps including the surgical consultation, during which the indication for surgery is determined, the preoperative consultation with the anesthesiologist and the ERAS nurse, hospital admission before surgery, the day of surgery, the stay in the intensive care unit, the stay in the intermediate care unit, and the postoperative ward stay before discharge. Throughout this pathway, several objectives are pursued: (1) patient education and engagement, including support and specific consultation for alcohol and tobacco cessation; (2) preoperative assessment of nutritional status using the Kondrup score and implementation of home-based nutritional support when indicated; (3) screening for frailty using the Clinical Frailty Scale and identification of impaired mobility, followed by targeted prehabilitation (preoperative outpatient or home-based physiotherapy). if needed; (4) blood management program with preoperative anemia screening and treatment; (5) bundle for infection prevention strategies; (6) reduction of the duration of sedation with the use of short acting agent (7) all effort to optimize invasive ventilation with systematic lung-protective ventilation strategies (Tidal volume of 6–8 ml/kg), use of positive end-expiratory pressure (≥ 5 cmH_2_O), recruitment maneuvers, early extubation followed by noninvasive ventilation; (8) goal-directed hemodynamic therapy; (9) optimization of pain management with an emphasis on opioid-sparing strategies; and (10) early mobilization with timely removal of invasive devices. Mobilization was defined as the patient getting out of bed, sitting in a chair, and taking a meal while seated. Mobilization progressively intensified with ambulation on a flat surface from postoperative day one, according to patient tolerance and clinical condition. Additional measures include prevention of hypothermia, delirium, and postoperative atrial fibrillation (POAF), alongside efforts to minimize operation and CPB duration, and ICU/hospital stay.

### Definition of postoperative pulmonary complications

PPCs were assessed throughout the entire postoperative hospital stay, from ICU admission to hospital discharge, including both ICU and ward periods, and were defined as follows. Respiratory infection was defined as the initiation of antibiotic therapy, by the clinician in charge, for a suspected pneumonia. Respiratory failure was defined by postoperative hypoxemia requiring supplemental oxygen therapy, non-invasive ventilation or reintubation. Pleural effusion was defined as a new or increased accumulation of fluid in the pleural space on chest imaging, characterized on chest radiograph by blunting of the costophrenic angle, loss of diaphragmatic contour, or displacement of adjacent anatomical structures, and on thoracic ultrasound as an anechoic or hypoechoic pleural fluid collection. Atelectasis was defined by clinical and radiological criteria of atelectasis with need for oxygen administration or non-invasive ventilation support and was defined as lung opacification associated with signs of volume loss on chest imaging, including mediastinal shift, displacement of the hilum or hemidiaphragm toward the affected side, and/or compensatory hyperinflation of adjacent non-atelectatic lung. Pneumothorax was defined as the presence of air in the pleural space requiring supplemental oxygen and/or pleural drainage. Bronchospasm was defined as the new onset of expiratory wheezing requiring bronchodilator treatment. Acute lung injury following the inhalation of regurgitated gastric contents was defined as aspiration pneumonitis. Imaging findings were interpreted as part of routine clinical care by the treating team, including intensivists and radiologists when applicable, without blinded adjudication. Each PPC component was recorded independently based on the predefined criteria, and multiple PPCs could be attributed to a single patient when clinically appropriate.

### Data collection

Baseline data included demographic characteristics, comorbidities, and functional status, encompassing age, sex, body mass index, smoking status, alcohol abuse, arterial hypertension, diabetes, dyslipidemia, chronic pulmonary and cerebrovascular disease, EuroSCORE II, left ventricular ejection fraction, symptomatic NYHA class, nutritional status, mobility, and frailty score. Surgical data comprised the type of cardiac procedure, operative duration, adherence to protective lung ventilation strategies, aortic cross-clamp time, cardiopulmonary bypass duration, need for transfusion and intraoperative opioid use. Postoperative variables included the duration of invasive mechanical ventilation and early extubation (≤ 6 h), opioid consumption (postoperative days 1 to 3, expressed as morphine milligram equivalents), postoperative blood loss, transfusion requirements, hemoglobin concentration on postoperative day 5, total intravenous fluid administration, and timing of postoperative mobilization. Postoperative course was further characterized by chest drain duration and the occurrence of major complications, including vasoplegia, right ventricular dysfunction, and the need for mechanical circulatory support. Data were collected prospectively after certification, using predefined variables established before protocol implementation. Clinical information was extracted from the hospital’s electronic medical records and entered into the REDCap (Research Electronic Data Capture) database.

### Objectives and outcomes

The primary objective of this study was to assess the incidence and the distribution of PPCs in patients managed under ERAS protocols. Secondary objectives were to explore modifiable perioperative and postoperative factors associated with PPCs and to assess the impact of PPCs on early postoperative course.

### Statistical analysis

Baseline patient characteristics and perioperative data were summarized for the entire cohort and compared patients with and without postoperative pulmonary complications (PPCs). Postoperative pulmonary complications were described according to their type, and the incidence of each complication was detailed. Clinical outcomes were analyzed by comparing patients with postoperative pulmonary complications to those without. Continuous variables were tested for normality using the Shapiro–Wilk test. Continuous variables were expressed as median (interquartile range) and compared using the Mann–Whitney *U* test. Categorical variables were expressed as counts (percentages) and compared using the *χ*^2^ test or Fisher’s exact test, as appropriate. Univariable logistic regression analyses were performed to identify potential factors associated with PPCs. Relevant variables were considered for inclusion in multivariable models. A primary multivariable logistic regression model was constructed including preoperative and intraoperative variables to identify independent risk factors. In a secondary exploratory analysis, selected postoperative variables were assessed in a separate model adjusted for baseline preoperative and intraoperative covariates. Missing data were handled by complete-case analysis; no imputation procedures were applied. Results are presented as odds ratios (OR) with their 95% confidence intervals (CI). All tests were two-tailed, and a *p*-value < 0.05 was considered statistically significant. Statistical analyses were performed using Stata version 18.0 (StataCorp LLC, College Station, TX, USA).

## Results

**Fig. 1:**
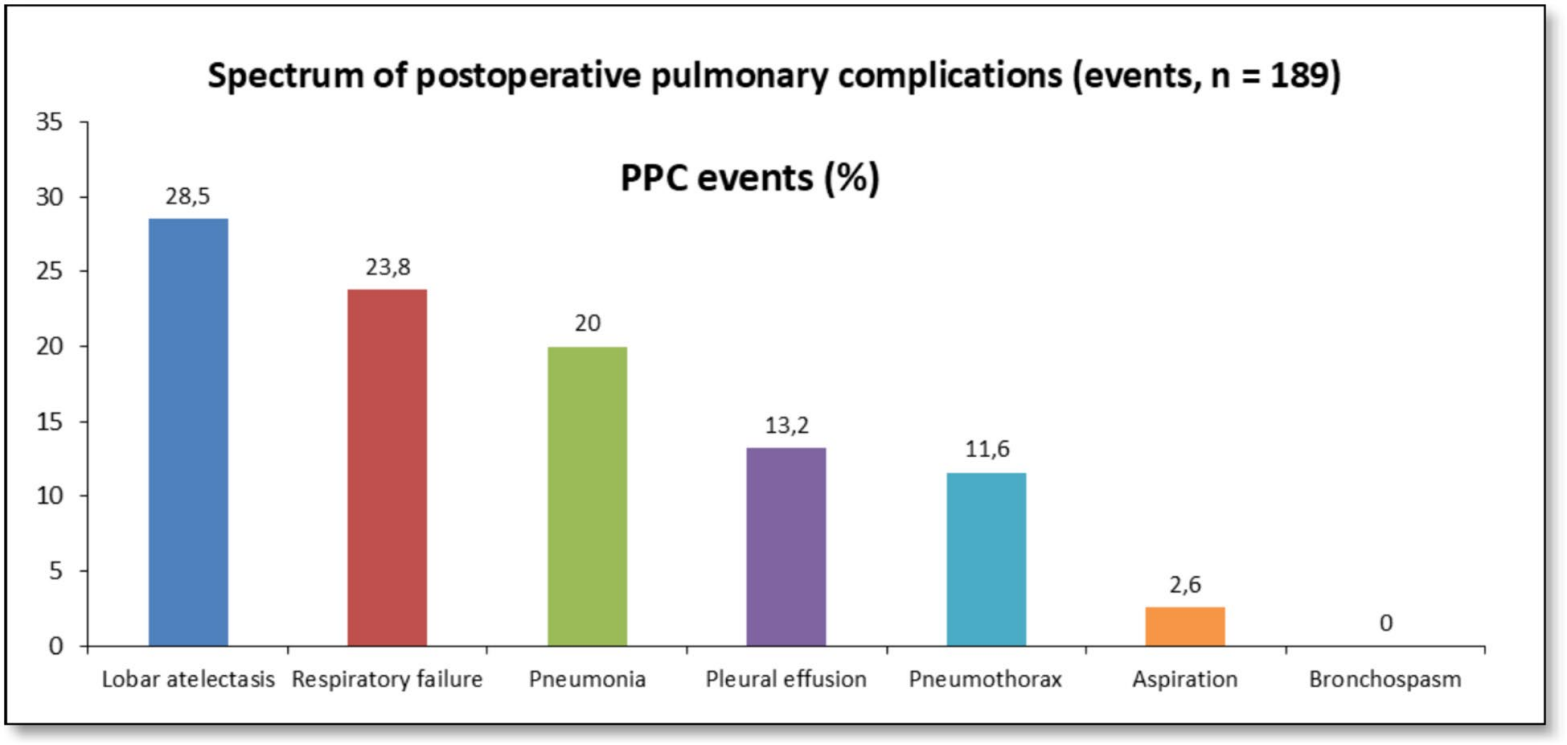
Distribution of postoperative pulmonary complications. Abbreviations: *PPC*, postoperative pulmonary complication

Among the 564 consecutive patients managed within the cardiac ERAS program during the study period, 64 were excluded: 42 declined consent for the use of their data, 14 had pending consent at the time of analysis, and 8 were excluded due to incomplete data. Consequently, 500 patients were included in the final analysis. Postoperative pulmonary complications (PPCs) occurred in 130 patients, with a total of 189 events recorded. Lobar atelectasis accounted for 54 events (28.5%), respiratory failure for 45 events (23.8%), pneumonia for 38 events (20.0%), pleural effusion for 25 events (13.2%), and pneumothorax for 22 events (11.6%). Aspiration of gastric contents was reported in 5 events (2.6%), and no cases of bronchospasm were observed (Fig. 1).

### Baseline characteristics and univariable associations with postoperative pulmonary complications

Among the 500 patients, those who developed PPCs were significantly older (67 (57–74) vs. 64 (55–71) years; *p* = 0.044), had a higher body mass index ≥ 35 kg/m^2^ (16% vs. 9%; *p* = 0.028), and had higher EuroSCORE II values (2.0 (1.0–3.5) vs. 1.4 (0.8–2.7); *p* = 0.038). PPCs were also significantly higher in redo surgery (16% vs. 8%; *p* = 0.008), ASA class III–IV (95% vs. 88%; *p* = 0.049), active smokers (29% vs. 17%; *p* = 0.011), chronic pulmonary disease (24% vs. 13%; *p* = 0.014), and severe left ventricular dysfunction (8% vs. 2%; *p* = 0.004). Regarding frailty, a Clinical Frailty Score ≥ 4 was more common in patients with PPCs (20% vs. 6%; *p* = 0.001), whereas scores 1–2 were less frequent in this group (21% vs. 40%; *p* = 0.001).

There were no significant differences between groups with respect to sex, body mass index (as a continuous variable), type of surgery, NYHA functional class, diabetes, hypertension, dyslipidemia or pulmonary arterial hypertension (Table 1).

**Table 1:** Baseline patient characteristics according to postoperative pulmonary complications

Variable	All patients (*n* = 500)	PPCs (*n* = 130)	No PPCs (*n* = 370)	*P *value
Age, years	65 (55–72)	67 (57–74)	64 (55–71)	0.044
Age ≥ 70 years	166 (33)	55 (42)	111 (30)	0.010
Male sex, *n* (%)	376 (75)	93 (71)	283 (76)	0.261
BMI, kg/m^2^	26 (20–30)	26 (23–30)	26 (24–30)	0.998
BMI ≥ 35 kg/m^2^, *n* (%)	58 (11)	22 (16)	36 (9)	0.028
EuroSCORE II	1.59 (0.9–2.9)	2.0 (1.0–3.5)	1.4 (0.8–2.7)	0.038
Type of surgery, *n* (%)				0.993
CABG	144 (29)	34 (26)	110 (30)	0.439
Valve surgery	192 (38)	45 (34)	147 (39)	0.302
Aortic surgery	31 (6)	10 (8)	21 (6)	0.412
Combined surgery	133 (26)	41 (31)	92 (25)	0.170
Redo surgery, *n* (%)	50 (10)	21 (16)	29 (8)	0.008
NYHA class, *n* (%)				0.319
I	149 (30)	32 (25)	117 (32)	
II	146 (30)	44 (34)	102 (28)	
III	92 (19)	28 (22)	64 (18)	
IV	8 (2)	3 (2)	5 (1)	
ASA class III–IV, *n* (%)	451 (90)	123 (95)	328 (88)	0.049
Diabetes, *n* (%)	97 (19)	21 (16)	76 (21)	0.277
Hypertension, *n* (%)	330 (67)	92 (71)	228 (65)	0.549
Dyslipidemia, *n* (%)	305 (62)	85 (65)	220 (61)	0.253
Current smokers, *n* (%)	101 (20)	38 (29)	63 (17)	0.011
Smoking cessation before surgery, *n* (%)	20 (4)	6 (5)	14 (4)	0.677
Chronic pulmonary disease, *n* (%)	79 (16)	31 (24)	48 (13)	0.014
Pulmonary arterial hypertension, *n* (%)	35 (7)	12 (9)	23 (6)	0.247
LVEF, %	60 (55–65)	60 (55–65)	60 (55–65)	0.885
Severe LV dysfunction, *n* (%)	18 (4)	10 (8)	8 (2)	0.004
Clinical Frailty Score, *n* (%)				0.001
1—very fit	36 (8)	6 (5)	30 (9)	
2—fit	126 (27)	20 (16)	106 (31)	
3—managing well	221 (48)	65 (53)	156 (45)	
4—living with very mild frailty	37 (8)	22 (18)	15 (4)	
≥ 5—frailty	10 (2)	3 (2)	7 (2)	
Clinical Frailty Score ≥ 4, *n* (%)	47 (11)	25(22)	22 (7)	0.001
Anemia screening and management *n* (%)	473 (94)	122 (94)	351 (95)	0.137
Preoperative nutritional status assessment *n* (%)	448 (90)	122 (94)	326 (89)	0.130
Preoperative physiotherapy *n* (%)	37 (7)	22 (17)	15 (4)	0.001

*Abbreviations*: *PPCs* postoperative pulmonary complications, *BMI* body mass index, *CABG* coronary artery bypass grafting, *NYHA* New York Heart Association, *ASA* American Society of Anesthesiologists, *LVEF* left ventricular ejection fraction, *LV* left ventricle, *PAH* pulmonary arterial hypertension, *CFS* Clinical Frailty Score

### Perioperative and postoperative characteristics and outcomes

PPCs were significantly associated with longer cardiopulmonary bypass duration and aortic cross-clamp duration (79 (61–117) vs. 72 (55–91) min; *p* = 0.006 and 60 (45–83) vs. 54 (40–70) min; *p* = 0.045, respectively). Duration of mechanical ventilation was longer (4.6 (0–19) vs. 0 (0–3.1) hours; *p* = 0.001), and early extubation within 6 h was less frequent (68% vs. 89%; *p* = 0.001) in the PPCs group. Opioid use was higher on postoperative day (POD) 2 (76% vs. 55%; *p* = 0.001) and POD 3 (40% vs. 19%; *p* = 0.001), as well as total opioid consumption (141 (29–276) vs. 100 (63–152) mg; *p* = 0.001). Patients with PPCs also experienced greater blood loss during the first 12 h (475 (321–800) vs. 410 (285–634) ml; *p* = 0.013), higher transfusion rates (41% vs. 24%; *p* = 0.001), lower hemoglobin levels on POD 5 (92 (85–103) vs. 98 (90–110) g/L; *p *= 0.001), and received larger volumes of total postoperative intravenous fluids (2.2 (1.0–3.7) vs. 1.3 (0.7–2.1) L; *p* = 0.001). Early mobilization was less frequent on POD 1 (82% vs. 95%; *p *= 0.001) and POD 2 (89% vs. 96%; *p* = 0.002), drain duration was longer (3 (2–4) vs. 2 (2–3) days; *p* = 0.001), and postoperative vasoplegia (20% vs. 8%; *p* = 0.001) and right ventricular dysfunction (7% vs. 2%; *p* = 0.011) were more frequent in the PPC group. No significant differences were observed between groups regarding protective lung ventilation, intraoperative opioid dose, opioid use on POD 1, mobilization on POD 0, and postoperative mechanical circulatory support (Table 2).

**Table 2:** Operative and postoperative course according to postoperative pulmonary complications

**Variable**	**All patients (*n* = 500)**	**PPCs (*n* = 130)**	**No PPCs (*n* = 370)**	***P* value**
CPB duration, min	74 (56–94)	79 (61–117)	72 (55–91)	0.006
Aortic cross-clamp duration, min	55 (41–73)	60 (45–83)	54 (40–70)	0.045
Protective lung ventilation, *n* (%)	479 (96)	124 (96)	355 (96)	0.775
IMV duration, h	0 (0–3.5)	4.6 (0–19)	0 (0–3.1)	0.001
Extubation ≤ 6 h, *n* (%)	415 (83)	89 (68)	326 (89)	0.001
OR extubation, *n* (%)	319 (63)	60 (46)	259 (70)	0.001
Intraoperative opioids, MME	35 (25–42)	32 (25–42)	35 (25–42)	0.943
Opioids on POD 1, *n* (%)	486 (98)	128 (98)	358 (98)	0.932
Opioids on POD 2, *n *(%)	300 (60)	99 (76)	201 (55)	0.001
Opioids on POD 3, *n* (%)	122 (25)	52 (40)	70 (19)	0.001
Total opioids, MME	110 (66–172)	141 (29–276)	100 (63–152)	0.001
Blood loss first 12 h, mL	425 (290–655)	475 (321–800)	410 (285–634)	0.013
Transfusion, *n* (%)	142 (28)	53 (41)	89 (24)	0.001
Hemoglobin POD5, g/L	96 (88–109)	92 (85–103)	98 (90–110)	0.001
Total postoperative IV fluids, L	1.5 (0.7–2.5)	2.2 (1.0–3.7)	1.3 (0.7–2.1)	0.001
Mobilization POD 0, *n* (%)	233 (47)	55 (42)	178 (49)	0.187
Mobilization POD 1, *n* (%)	458 (91)	107 (82)	351 (95)	0.001
Mobilization POD 2, *n* (%)	473 (95)	116 (89)	357 (96)	0.002
Drain duration, days	2 (2–3)	3 (2–4)	2 (2–3)	0.001
Postoperative vasoplegia, *n* (%)	55 (11)	26 (20)	29 (8)	0.001
Postoperative RV dysfunction, *n *(%)	16 (3)	9 (7)	7 (2)	0.011
Postoperative MCS, *n* (%)	4 (1)	2 (2)	2 (0.5)	0.111

*Abbreviations*: *PPCs* postoperative pulmonary complications, *CPB* cardiopulmonary bypass, *ACC* aortic cross-clamp, *IMV* invasive mechanical ventilation, *POD* postoperative day, *OR* operating room, *MME* morphine milligram equivalents, *IV* intravenous, *RV* right ventricle, *MCS* mechanical circulatory support

### Postoperative complications and outcomes

Patients with PPCs had significantly longer hospital and ICU length of stay (13 (9–17) vs. 8 (7–11) days; *p* = 0.001 and 2.0 (1.1–4.0) vs. 1.0 (0.9–1.8) days; *p* = 0.001, respectively), as well as higher rates of ICU readmission (10.0% vs. 1.9%; *p* = 0.001). Acute confusional state (14.6% vs. 3.0%; *p* = 0.001), acute kidney injury (10.8% vs. 3.5%; *p* = 0.002), other than pneumonia infectious complications (19.2% vs. 9.2%; *p* = 0.002), ICU-acquired weakness or neuropathy (2.3% vs. 0.3%; *p* = 0.015), and the occurrence of any complication other than PPCs (66.2% vs. 38.9%; *p* = 0.001) were all more frequent in patients with PPCs. Early reintubation (3.1% vs. 0.5%; *p* = 0.063) and postoperative atrial fibrillation (34.6% vs. 26.5%; *p* = 0.078) were more common in patients with PPCs but did not reach statistical significance. No significant differences were observed between groups regarding thirty-day mortality (2.3% vs. 0.8%; *p* = 0.178), re-operation (8.5% vs. 5.1%; *p* = 0.170), and deep venous thrombosis (1.5% vs. 0.3%; *p* = 0.111) (Table 3).

**Table 3:** Clinical outcomes according to postoperative pulmonary complications

Outcome	PPCs (*n* = 130)	No PPCs (*n* = 370)	*P* value
30-day mortality, *n* (%)	3 (2.3)	3 (0.8)	0.178
Hospital length of stay, days	13 (9–17)	8 (7–11)	0.001
ICU length of stay, days	2.0 (1.1–4.0)	1.0 (0.9–1.8)	0.001
ICU readmission, *n* (%)	13 (10.0)	7 (1.9)	0.001
Early re-intubation, *n* (%)	4 (3.1)	2 (0.5)	0.063
Re-operation, *n* (%)	11 (8.5)	19 (5.1)	0.170
Acute confusional state, *n* (%)	19 (14.6)	11 (3.0)	0.001
Acute kidney injury, *n* (%)	14 (10.8)	13 (3.5)	0.002
Infectious complications, *n* (%)	25 (19.2)	34 (9.2)	0.002
Postoperative atrial fibrillation, *n* (%)	45 (34.6)	98 (26.5)	0.078
ICU-acquired weakness/neuropathy, *n* (%)	3 (2.3)	1 (0.3)	0.015
Deep venous thrombosis, *n* (%)	2 (1.5)	1 (0.3)	0.111
Deep wound dehiscence, *n* (%)	1 (0.8)	0 (0.0)	0.093
Any complication, *n* (%)	86 (66.2)	144 (38.9)	0.001

*Abbreviations*: *PPCs* postoperative pulmonary complications, *ICU* intensive care unit, *AKI* acute kidney injury, *POAF* postoperative atrial fibrillation

Frailty was identified in 10.9% of patients (*n* = 47) and was associated with a significantly higher incidence of postoperative respiratory complications (53.2% vs 23.8% in non-frail patients, *p* < 0.001). Among the cohort, 430 (86%) patients were screened for frailty (missing data for 70 patients). Frail patients more frequently received targeted preoperative interventions, including nutritional treatment, physiotherapy consultation, and prehabilitation; detailed comparisons between frail and non-frail patients are provided in Table 4.

**Table 4:** Comparison of postoperative respiratory complications and preoperative interventions between frail and non-frail patients

Variable	Non-frail (*n* = 383)	Frail (*n* = 47)	*p* value
Postoperative respiratory complication	91 (23.8%)	25 (53.2%)	< 0.001
Preoperative nutritional treatment	19 (5.0%)	8 (17.0%)	0.001
Preoperative nutritional status assessment	373 (97.4%)	45 (95.7%)	0.52
Physiotherapy consultation	1 (0.3%)	34 (72.3%)	< 0.001
Prehabilitation program	6 (1.6%)	12 (25.5%)	< 0.001

### Multivariable analysis

**Fig. 2:**
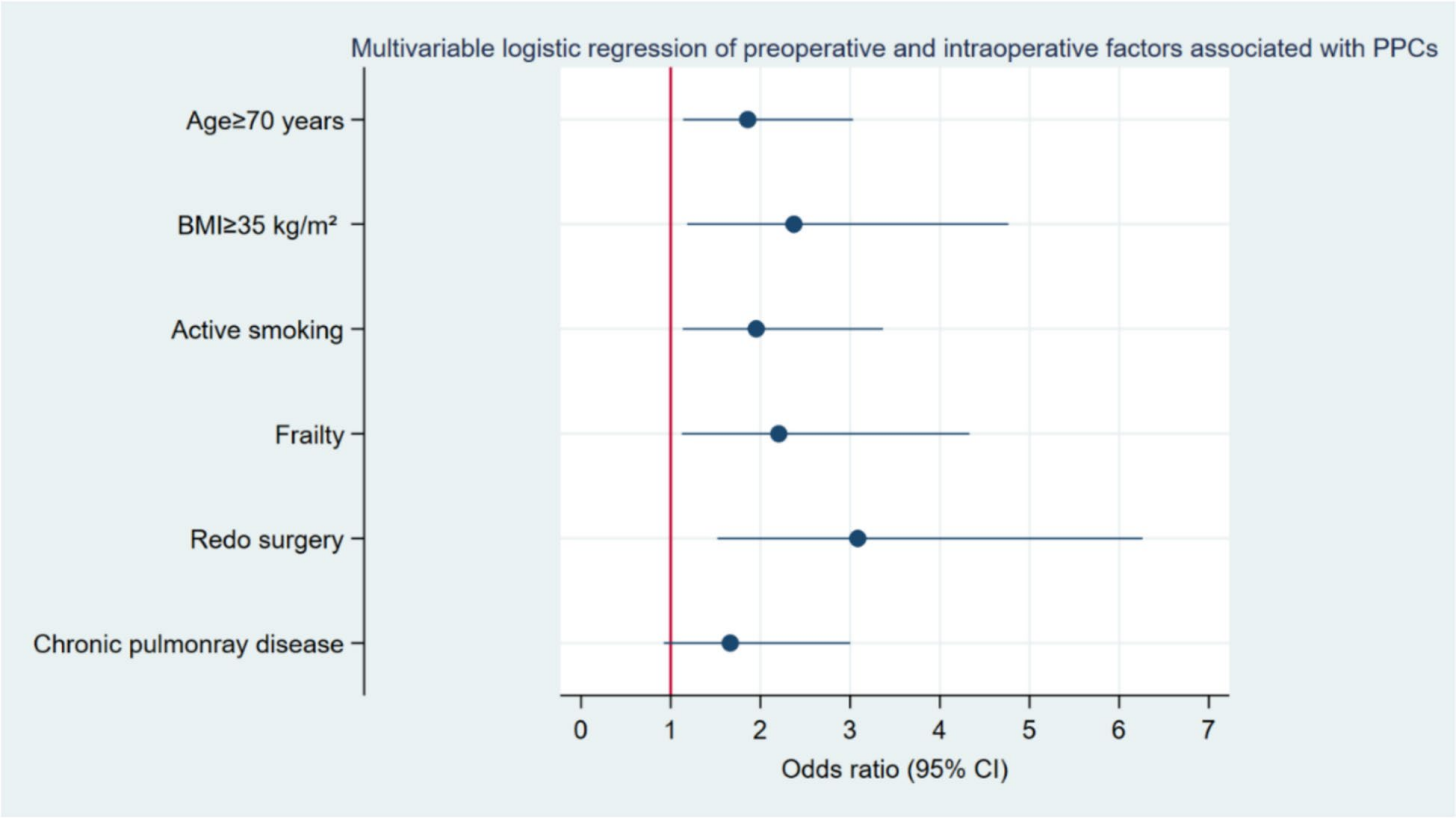
Multivariable logistic regression analysis of preoperative and intraoperative factors associated with postoperative pulmonary complications (PPCs). Adjusted odds ratios (ORs) with 95% confidence intervals are displayed for preoperative variables (age ≥ 70 years, BMI ≥ 35 kg/m^2^, active smoking, frailty, and chronic pulmonary disease) and the intraoperative variable (redo surgery). The vertical red line represents the null value (OR = 1). Abbreviations: PPCs, postoperative pulmonary complications; BMI, body mass index; CI, confidence interval; OR, odds ratio

In the primary multivariable model including preoperative and intraoperative variables (complete-case analysis, *n* = 430, missing data 14%), age ≥ 70 years, obesity (BMI ≥ 35 kg/m^2^), active smoking, frailty (CFS ≥ 4), and redo surgery were independently associated with the occurrence of PPCs. Specifically, age ≥ 70 years (OR 1.86, 95% CI 1.14–3.03, *p* = 0.013), BMI ≥ 35 kg/m^2^ (OR 2.37, 95% CI 1.18–4.76, *p* = 0.015), active smoking (OR 1.95, 95% CI 1.13–3.37, *p* = 0.016), and frailty (OR 2.21, 95% CI 1.12–4.33, *p* = 0.022) were associated with approximately a twofold increase in the odds of PPCs. Redo surgery was strongly associated with PPCs (OR 3.09, 95% CI 1.52–6.26, *p* = 0.002). Chronic pulmonary disease showed a non-significant trend toward increased risk (OR 1.66, 95% CI 0.92–3.00, *p* = 0.090) (Table 5, Fig. 2).

**Table 5:** Multivariable logistic regression analysis of preoperative and intraoperative factors associated with PPCs

Variable	Adjusted OR	95% CI	*p*-value
Age ≥ 70 years	1.86	1.14–3.03	0.013
BMI ≥ 35 kg/m^2^	2.37	1.18–4.76	0.015
Active smoking	1.95	1.13–3.37	0.016
Frailty (CFS ≥ 4)	2.21	1.12–4.33	0.022
Redo surgery	3.09	1.52–6.26	0.002
Chronic pulmonary disease	1.66	0.92–3.00	0.090

In exploratory multivariable logistic regression analysis including perioperative and postoperative factors (complete-case analysis, *n* = 430; missing data 14%), age ≥ 70 years was independently associated with an increased risk of postoperative pulmonary complications (adjusted OR 1.74, 95% CI 1.05–2.9; *p* = 0.033). Obesity (body mass index ≥ 35 kg/m^2^) was associated with higher odds of PPCs (adjusted OR 2.61, 95% CI 1.25–5.42; *p* = 0.010), as was active smoking (adjusted OR 2.04, 95% CI 1.16–3.60; *p* = 0.013). Frailty was associated with a threefold increase in risk of PPCs (adjusted OR 2.88, 95% CI 1.42–5.83; *p* = 0.003), while postoperative delirium was associated with the highest odds of PPCs (adjusted OR 4.13, 95% CI 1.59–10.72; *p* = 0.004). Blood transfusion was independently associated with an increased risk of postoperative pulmonary complications (adjusted OR 1.74, 95% CI 1.04–2.93; *p* = 0.036). Each additional day of drain duration increased the risk of PPCs by 17% (adjusted OR 1.17, 95% CI 1.03–1.32; *p* = 0.013). In contrast, early extubation within 6 h reduced the risk of PPCs by 60% (adjusted OR 0.40, 95% CI 0.21–0.75; *p* = 0.004) (Fig. S1 and Table S1 in Supplementary Material).

## Discussion

In this single-center cohort of 500 consecutive patients undergoing cardiac surgery within an established ERAS program, postoperative pulmonary complications (PPCs) occurred in 26% of patients. Atelectasis, respiratory failure, and pneumonia account for nearly 70% of the events. Previous reports in cardiac surgery describe a wide range of PPCs incidence, ranging from 3 to 55% [2, 13–16]. This large variability is mainly explained by substantial heterogeneity in patient characteristics and surgical complexity, as well as by differences in PPCs definitions. In a large contemporary cohort of 588,480 patients undergoing elective coronary artery bypass grafting and/or valve surgery, Hadaya et al. reported a relatively low PPCs incidence of 6.7%. In that study, PPCs were defined by the occurrence of at least one major event: reintubation, tracheostomy, prolonged mechanical ventilation, or pneumonia, thereby excluding several common but clinically relevant pulmonary complications such as atelectasis, pleural effusion, or pneumothorax, which likely explains the low reported incidence [15].

In contrast, the VENICE international multicenter study, published by Fischer et al. in 2022, adopted a broader, more comprehensive definition of PPCs based on the 2015 European Task Force guidelines for perioperative clinical outcome definitions (EPCO) [17]. Using criteria similar to those applied in our cohort, including several minor and major pulmonary events, the authors reported that 55% experienced at least one PPC during follow-up [2]. Similar incidences were reported by Liu et al. and Wang et al., with rates of 56.85% and 51.6%, respectively, using the EPCO criteria. Although PPCs’ definitions were broadly comparable to those used in VENICE, we observed a lower incidence in our cohort. Our cohort consisted exclusively of elective surgeries and was characterized by relatively low EuroSCORE II values, which may partly explain the lower incidence observed. Nevertheless, this does not diminish the potential impact of implementing a structured ERAS pathway emphasizing fast-track recovery, including extubation in the operating room or within the first hours after surgery, systematic protective lung ventilation, systematic non-invasive ventilation after extubation, and opioid-sparing analgesia, all of which directly target mechanisms leading to PPCs. In addition, restrictive perioperative fluid administration and timely chest drain removal after early mobilization may have contributed by limiting pulmonary congestion and promoting the recovery of an effective cough and lung expansion, as reflected by the lower incidence of pleural effusion in our cohort (13%).

Several non-modifiable factors associated with PPCs were identified in our cohort, including older age, obesity (BMI ≥ 35 kg/m^2^), and a higher baseline surgical risk as reflected by elevated EuroSCORE II values. In addition, factors reflecting patient comorbidity and procedural complexity, such as redo surgery, ASA class III–IV, chronic pulmonary disease, and severe left ventricular dysfunction, have consistently been associated with an increased PPCs risk in cardiac surgery [18]. In the multivariable analysis focusing on preoperative and intraoperative variables, older age (> 70 years), obesity (BMI > 35), active smoking, frailty, and redo surgery were independently associated with PPCs. Active smoking was more frequent among patients who developed PPCs, confirming smoking as an important factor linked to postoperative pulmonary complications. In contrast, preoperative smoking cessation was not associated with a reduced risk of PPCs in our cohort. This may be explained by the limited number of patients who accepted a dedicated smoking cessation consultation (only 20 patients) and by the short interval between consultation and surgery, which was generally shorter than the 4-week period shown in the literature to confer pulmonary benefit [19]. This low adherence likely reflects real-world implementation challenges, including short preoperative timelines limiting access to structured interventions, as well as suboptimal patient adherence to smoking cessation programs. But it also underscores a major gap in the application of ERAS recommendations. Strengthening structured smoking cessation pathways should therefore be considered a key target for quality improvement in future iterations of our program.

In our cohort, frailty was present in 10.9% of patients and was strongly associated with postoperative respiratory complications. More than half of frail patients (53.2%) developed a respiratory complication. Importantly, frailty remained an independent predictor of postoperative respiratory complications after multivariable adjustment, confirming its robust prognostic impact beyond traditional clinical and perioperative risk factors. This finding is consistent with previous literature reporting odds ratios exceeding four in some cohorts [20–22]. Frailty reflects a multidimensional state of vulnerability, combining impaired physical reserve, poor mobility, sarcopenia, and nutritional deficits, and represents a potentially modifiable factor [23]. In our practice, identification of frailty was associated with targeted perioperative interventions. Nearly all frail patients (97%) received a preoperative nutritional status assessment. Frail patients were also more likely to receive preoperative nutritional treatment (17.0% vs 5.0%), physiotherapy consultation (72.3% vs 0.3%), and ambulatory prehabilitation (25.5% vs 1.6%). Taken together, these findings indicate that while frailty is adequately identified in our cohort, rehabilitative and nutritional strategies remain underutilized. The relatively low prevalence of frailty observed in our cohort may reflect, at least in part, a selection bias, as patients with advanced frailty and prohibitive surgical risk may have been excluded during preoperative evaluation. Alternatively, under-recognition of frailty or specific characteristics of our surgical population may also contribute. Despite systematic screening, prehabilitation uptake remained limited (25.5% of frail patients), likely reflecting a combination of short preoperative timelines, suboptimal patient adherence, and logistical or resource constraints. These findings highlight an important gap between frailty identification and implementation of corrective strategies. Future efforts should focus on earlier identification during surgical consultation and the development of structured and streamlined prehabilitation pathways.

Redo surgery was independently associated with PPCs, likely reflecting increased surgical complexity and prolonged operative times. Repeat sternotomy is typically associated with greater technical difficulty, higher bleeding risk, increased transfusion requirements, more extensive tissue dissection, and higher postoperative morbidity and mortality [24]. Furthermore, longer cardiopulmonary bypass duration and delayed recovery frequently observed in redo procedures may further increase the risk of respiratory complications, in line with previous studies highlighting the impact of surgical complexity and intraoperative factors on PPC occurrence [25].

We performed an exploratory multivariable analysis to assess potential postoperative factors associated with PPCs, adjusted for preoperative and intraoperative variables. Early extubation, postoperative delirium, transfusion, and chest drain duration were identified as significant associations. Early extubation was associated with a lower incidence of PPCs, consistent with current ERAS and Society of Thoracic Surgeons recommendations supporting fast-track strategies [9]. Early extubation limits exposure to mechanical ventilation, a well-recognized risk factor for ventilator-associated pulmonary complications such as pneumonia and atelectasis. While reduced exposure to mechanical ventilation may limit ventilator-associated complications, delayed extubation may also reflect intraoperative complexity or early postoperative instability predisposing to delayed extubation and PPCs. In our cohort, median duration of thoracic drainage was two days, and duration was associated with PPCs with each additional day increasing PPC risk by 17%. Consistent with previous evidence and guidelines, early chest tube removal within an ERAS protocol is safe and associated with reduced bronchopneumonia, shorter ICU and hospital length of stay, and earlier mobilization, without increasing pneumothorax, effusions requiring intervention, or hospital mortality [9, 26, 27]. The relation between early drain removal and PPCs is likely bidirectional, as prolonged drainage may contribute to impaired respiratory mechanics and delayed mobilization, while PPCs or clinical instability may themselves delay drain removal. Postoperative delirium also emerged in our cohort as a strong and independent correlate of PPCs. In many studies, delirium has been associated with prolonged mechanical ventilation and postoperative pneumonia [28, 29]. Also, delirium further increases PPC risk by impairing cooperation with respiratory physiotherapy, effective coughing, and early mobilization. However, causality is difficult to establish and the relationship between delirium and PPCs is likely bidirectional, as delirium may impair respiratory recovery, while PPCs may also precipitate delirium through hypoxemia, inflammation and prolonged sedative exposure [30]. Our study cannot infer causality in this cause-and-effect relationship, but our findings nonetheless support integrating systematic delirium screening, prevention, and early management as a core component of the ERAS pathway to prevent PPCs.

Although anemia screening and management were implemented in most of our cohort (94%), reflecting strong adherence to patient blood management, perioperative transfusion exposure remained substantial, occurring in 28% of the overall cohort and in more than 40% of patients who developed PPCs. Transfusion was independently associated with significantly increased postoperative pulmonary complications, with a 74% relative increase in risk. This finding is consistent with previous literature identifying transfusion as a major modifiable risk factor for postoperative respiratory distress, respiratory failure, longer intubation times, acute respiratory distress, reintubation and pneumonia after cardiac surgery [31, 32]. In our institution, a restrictive transfusion strategy is applied, with a hemoglobin threshold of 70 g/L, except in the presence of clinical signs of inadequate tissue oxygenation, or active heart failure or myocardial ischemia, in which case higher thresholds (90 g/L) may be considered. These situations are relatively frequent in the postoperative cardiac surgery setting. We did not collect detailed data regarding transfusion triggers or indications, limiting further interpretation of this finding. Importantly, our results should be interpreted with caution, as transfusion exposure and postoperative hemoglobin levels may reflect underlying patient severity, perioperative bleeding, or surgical complexity rather than transfusion practice alone. Accordingly, transfusion may represent both a potential contributor to PPCs and a marker of more severe clinical conditions.

All the aforementioned variables (early extubation, transfusion, drain removal and delirium) occur in the postoperative period, a causal relationship cannot be firmly established, and these associations are likely bidirectional. These factors may contribute to the development of PPCs but may also reflect underlying patient severity or arise as a consequence of evolving respiratory complications. Therefore, these findings should be interpreted as hypothesis-generating and may highlight potential targets for quality improvement within ERAS pathways.

Our study suggests that patients who developed PPCs experienced a more complicated postoperative course, with longer durations of mechanical ventilation and increased ICU and hospital length of stay, consistent with prior reports in cardiac surgery [2, 15, 33]. We did not observe a significant difference in mortality, likely because our ERAS cohort predominantly included low-risk elective cardiac surgery patients, resulting in low overall mortality and limiting our ability to detect an excess mortality attributable to PPCs.

Several limitations should be acknowledged. The single-center design may limit the generalizability of the findings to other institutions with different surgical case mixes, patient populations and perioperative practice. However, we standardized ERAS pathway and perioperative management which strengthen our finding and internal validity. Second, the observational nature of the study precludes causal inference despite multivariable adjustment and observed associations may reflect reverse causality, whereby patients developing early PPCs are more likely to experience prolonged ventilatory support or delayed drain removal. Third, PPCs represent a heterogenous composite of different respiratory events with different pathophysiology, risk factors. However, we aim to remain aligned with commonly used perioperative outcome definitions, even if this approach may obscure differences across individual PPC subtypes. Fourth, detailed perioperative ventilatory parameters were not specifically analyzed in our data and deviations in intraoperative and postoperative respiratory management may not have been fully accounted for. Nevertheless, lung-protective ventilatory strategies were protocolized at our institution and represent the standard of care for ERAS cardiac surgery patients. Fifth, this study reflects the early experience of an ERAS cardiac surgery program, and temporal changes in team adherence, learning effects, and protocol refinement over time may have influenced outcomes and larger multicenter cohorts focusing on specific ERAS components are warranted to confirm these findings and better describe causal pathways. Sixth, although our PPCs composite was aligned with the EPCO framework, several components were defined in a more treatment-driven manner. While these pragmatic definitions may enhance clinical relevance by capturing events requiring therapeutic intervention, they may also lead to differences in event classification and limit comparability with studies strictly adhering to EPCO criteria. Finally, although a historical pre-ERAS cohort was available at our institution, the retrospective nature of its documentation raised concerns regarding the consistency of PPC definitions and the completeness of data collection. To preserve internal validity and ensure robust and standardized outcome assessment, we deliberately restricted the analysis to the ERAS cohort. As a result, no direct before-after comparison of ERAS implementation could be performed, and the potential impact of the ERAS pathway on PPC incidence cannot be established. Accordingly, our findings should be interpreted as descriptive and hypothesis-generating.

## Conclusion

In summary, postoperative pulmonary complications occurred in approximately one in four patients in our ERAS cardiac surgery cohort, with the most frequent events being atelectasis, respiratory failure, and pneumonia. Our findings highlighted the importance of several preoperative factors associated with PPCs, particularly advanced age, obesity, active smoking, and frailty. Among these, frailty and active smoking may represent potentially modifiable factors that remain insufficiently addressed and could be targets for future improvement. In addition, exploratory analyses identified several postoperative factors, including early extubation, delirium, transfusion, and chest drain management, as potential targets for optimization. Further studies are needed to better clarify their causal role and to guide targeted interventions.

## Supplementary Information

Supplementary Material 1. Table S1. Multivariable logistic regression analysis for postoperative respiratory complications. Figure S1. Multivariable Logistic Regression Analysis of Postoperative Pulmonary Complications

## Data Availability

All data generated or analyzed during this study are included in this published article.

## Abbreviations

ACC: Aortic cross-clamp
AKI: Acute kidney injury
ASA: American Society of Anesthesiologists
BMI: Body mass index
CABG: Coronary artery bypass grafting
CFS: Clinical Frailty Scale
CPB: Cardiopulmonary bypass
ICU: Intensive care unit
IMV: Invasive mechanical ventilation
IV: Intravenous
LV: Left ventricle
LVEF: Left ventricular ejection fraction
MCS: Mechanical circulatory support
MME: Morphine milligram equivalents
NYHA: New York Heart Association
OR: Operating room
PAH: Pulmonary arterial hypertension
POD: Postoperative day
POAF: Postoperative atrial fibrillation
PPCs: Postoperative pulmonary complications
RV: Right ventricle
