# Supplementary material for: Incidence, patterns, and factors associated with postoperative pulmonary complications in an ERAS cardiac surgery program: a 500-patient cohort study
Source: J Anesth Analg Crit Care. 2026 May 8;6:91. doi: 10.1186/s44158-026-00402-x (PMC13321419; doi:10.1186/s44158-026-00402-x)
Supplement: Supplementary file 1 — Supplementary Material 1. Table S1. Multivariable logistic regression analysis for postoperative respiratory complications. Figure S1. Multivariable Logistic Regression Analysis of Postoperative Pulmonary Complications [file 44158_2026_402_MOESM1_ESM.docx]

**Supplementary Material**

**Table s1.** Multivariable logistic regression analysis for postoperative respiratory complications

| **Variable** | **Adjusted OR** | **95% CI** | **p value** |
| --- | --- | --- | --- |
| Age ≥70 years | 1.74 | 1.05–2.90 | 0.033 |
| BMI ≥35 kg/m² | 2.61 | 1.25–5.42 | 0.010 |
| Active smoking | 2.04 | 1.16–3.60 | 0.013 |
| Frailty | 2.88 | 1.42–5.83 | 0.003 |
| Chest drain duration (per day) | 1.17 | 1.03–1.32 | 0.013 |
| Early extubation ≤6 h | 0.40 | 0.21–0.75 | 0.004 |
| Postoperative delirium | 4.13 | 1.59–10.72 | 0.004 |
| Any transfusion | 1.74 | 1.04–2.93 | 0.036 |

**Abbreviations:** OR, odds ratio; CI, confidence interval; BMI, body mass index; CPB, cardiopulmonary bypass.

**Figure s1.** Multivariable Logistic Regression Analysis of Postoperative Pulmonary Complications

**
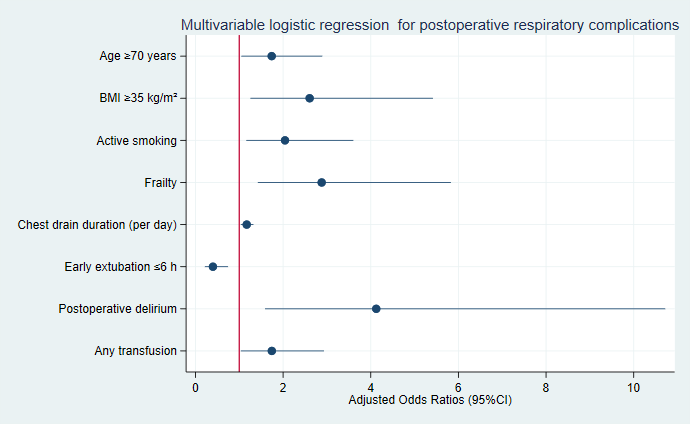
**

Figure s1. Multivariable analysis of factors associated with postoperative pulmonary complications (PPCs). Adjusted odds ratios (ORs) with 95% confidence intervals are presented for preoperative risk factors (age ≥70 years, BMI ≥35 kg/m², active smoking, frailty), intraoperative factors (redo surgery), and postoperative variables (early extubation, delirium, transfusion, and chest drain duration).

**Abbreviations:** PPCs, postoperative pulmonary complications; OR, odds ratio; CI, confidence interval; BMI, body mass index; CFS, Clinical Frailty Scale.
